# Response of sinusoidal mouse liver cells to choline-deficient ethionine-supplemented diet

**DOI:** 10.1186/1476-5926-9-8

**Published:** 2010-10-13

**Authors:** Elke Ueberham, Jan Böttger, Uwe Ueberham, Jens Grosche, Rolf Gebhardt

**Affiliations:** 1 Institute of Biochemistry, Medical Faculty, University of Leipzig, Leipzig, Germany; 2 Department for Molecular and Cellular Mechanisms of Neurodegeneration, University of Leipzig, Paul Flechsig Institute of Brain Research, Leipzig, Germany; 3 Department for Pathophysiology of Neuroglia, University of Leipzig, Paul Flechsig Institute of Brain Research, Leipzig, Germany; 4 Interdisciplinary Centre for Clinical Research, Medical Faculty of the University of Leipzig, Leipzig, Germany

## Abstract

**Background:**

Proliferation of oval cells, the bipotent precursor cells of the liver, requires impeded proliferation and loss of hepatocytes as well as a specific micro-environment, provided by adjacent sinusoidal cells of liver. Despite their immense importance for triggering the oval cell response, cells of hepatic sinusoids are rarely investigated. To elucidate the response of sinusoidal liver cells we have employed a choline-deficient, ethionine-supplemented (CDE) diet, a common method for inducing an oval cell response in rodent liver. We have utilised selected expression markers commonly used in the past for phenotypic discrimination of oval cells and sinusoidal cells: cytokeratin, E-cadherin and M2-pyruvate kinase for oval cells; and glial fibrillary acidic protein (GFAP) was used for hepatic stellate cells (HSCs).

**Results:**

CDE diet leads to an activation of all cells of the hepatic sinusoid in the mouse liver. Beside oval cells, also HSCs and Kupffer cells proliferate. The entire fraction of proliferating cells in mouse liver as well as endothelial cells and cholangiocytes express M2-pyruvate kinase. Concomitantly, GFAP, long considered a unique marker of quiescent HSCs was upregulated in activated HSCs and expressed also in cholangiocytes and oval cells.

**Conclusions:**

Our results point to an important role of all types of sinusoidal cells in regeneration from CDE induced liver damage and call for utmost caution in using traditional marker for identifying specific cell types. Thus, M2-pyruvate kinase should no longer be used for estimating the oval cell response in mouse liver. CDE diet leads to activation of GFAP positive HSCs in the pericentral zone of liver lobulus. In the periportal zone the detection of GFAP in biliary cells and oval cells, calls other cell types as progenitors of hepatocytes into question under CDE diet conditions.

## Background

Oval cell reaction occurs under pathological conditions in human liver and in early stages of experimental hepatocarcinogenesis protocols in rodents provided hepatocyte proliferation is impaired. A frequently used protocol applies ethionine, the ethyl analogon of methionine, together with a choline deficient diet (CDE) [1]. During CDE diet many metabolic changes in hepatocytes take place leading to deposition of lipids in hepatocytes and massive lethal deterioration of this cell type. Surviving hepatocytes are no longer able to proliferate and to repopulate the damaged tissue. Instead, oval cells, the bipotential progenitor cells of liver that are resistant against the destroying mechanisms, are activated and enrich. For proliferation they require a typical microenvironment which is provided by cells of the hepatic sinusoids closely adjacent to them. The pivotal role of an intrahepatic inflammatory response in this process, and the recruitment of Kupffer cells and other intrahepatic leukocytes were recently described in CDE treated mice [2,3]. In addition to macrophages and monocytes other cells of hepatic sinusoids also contribute to this environment as it was recently shown for myofibroblasts [4]. Changes concerning sinusoidal cells under CDE conditions are rarely investigated until now. An increase of the non-hepatocytic pyruvate kinase was demonstrated, however, in livers of CDE treated mice [2,5,6].

In adult liver, different isoenzymes of pruvate kinase (Pk) exist. The L-isoenzyme is exclusively expressed in hepatocytes (L-Pk) [7,8], whereas the M-isoenzyme (M-Pk) occurs in sinusoidal cells. From M-Pk two splice variants, the M1-Pk and M2-Pk, were detected. M2-Pk, known as the embryonic or tumor type, also belongs to the normal enzymatic configuration of cholangiocytes, hepatic stellate cells (HSCs) [9] and Kupffer cells [10] of rat liver. A switch from M1- to M2-type was demonstrated in rapidly growing cells [11], and M2-type was found to be expressed in oval cells [12,13]. Although M2-Pk was detected in most sinusoidal cell types in rat liver, it has gained the status of an oval cell marker particularly in mouse [5,6,14,15]. However, the distribution of Pk isoenzymes among mouse sinusoidal cells has not been explicitly studied yet.

In the present study, we dissected the response of sinusoidal cells in the liver of CDE treated mice. We verified that CDE diet provokes enrichment and/or activation of all sinusoidal cells, and show that M2-Pk is expressed in nearly all cells of hepatic sinusoids in mouse liver except of smooth muscle cells and myofibroblasts. Thus, M-Pk cannot be used as a reliable marker of oval cells. Additionally, we found an overlapping expression of glial fibrillary acidic protein (GFAP) in epithelial (cholangiocytes, oval cells) and mesenchymal (HSCs) cells of mouse liver, rendering this marker useless for unequivocally tracing precursor cell lineages.

## Results

### M-Pk signal is not an oval cell specific response

**Figure 1:**
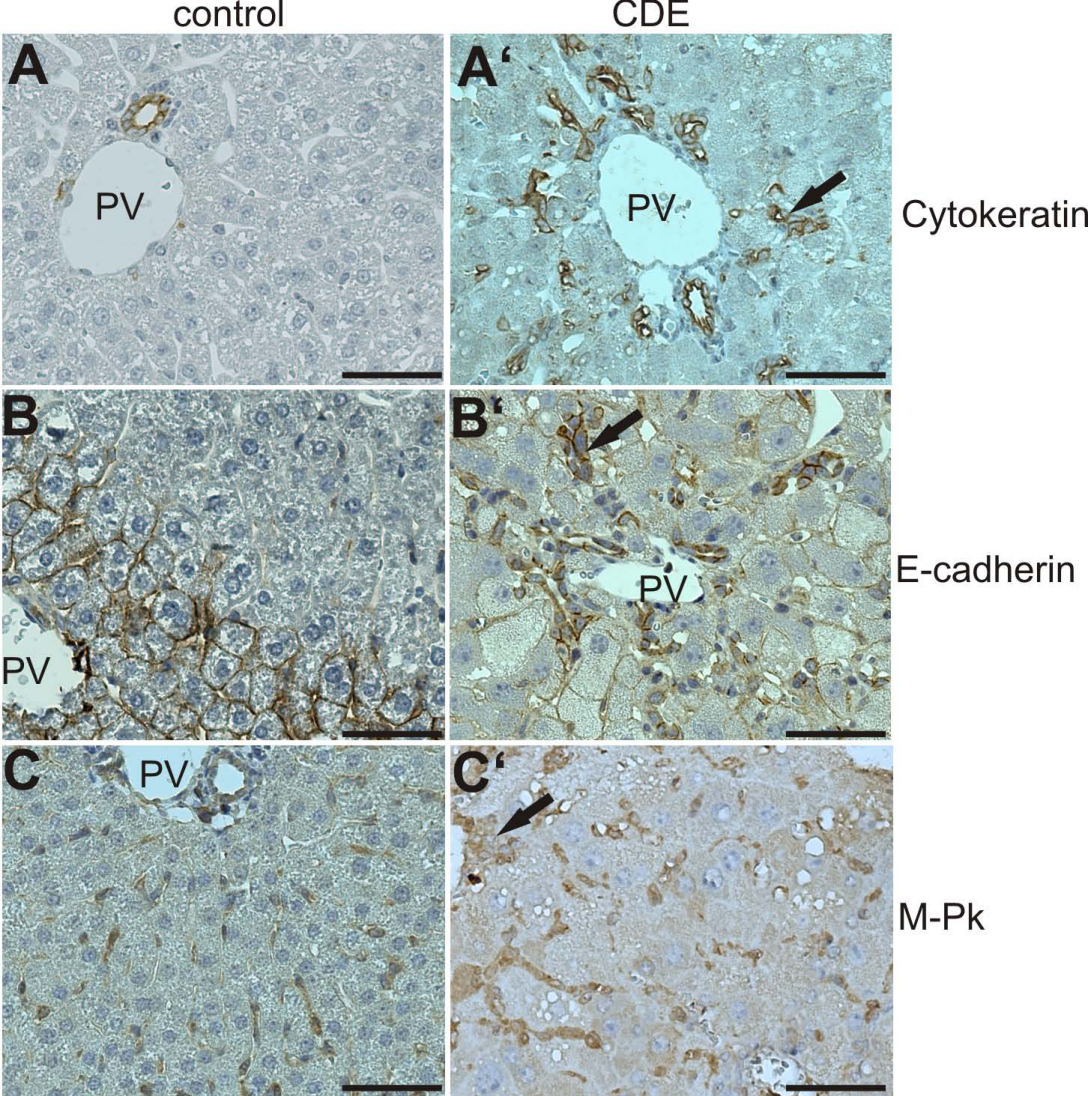
**CDE diet induces both an oval cell response and a response of sinusoidal liver cells**. Immunohistochemical stainings of cytokeratin, E-cadherin and M-Pk were compared from normal mice (left panel) and CDE treated mice (right panel). Black arrows indicate ductular accumulation of oval cells. These cells were displayed with a pan specific anti-cytokeratin antibody (A, A'). This antibody additionally detects cells of biliary ducts. An immunohistochemical staining with anti-E-cadherin antibody reliably displays oval cells, but reacts also with biliary cells and additionally with periportal hepatocytes. The anti-M-Pk antibody (Rockland, Table 1) marks oval cells but also biliary cells and cells of hepatic sinusoids. Sinusoidal cells accumulate under CDE conditions (C') PV = portal vein. Bar = 50 μm.

We used the CDE diet protocol to induce an oval cell response and proved the hypothesis that M-Pk is convenient to scale this oval cell reaction. To examine the effectiveness of our diet conditions, we determined E-cadherin levels, previously found strongly elevated during CDE diet [4] and also indicating a strong oval cell response [16]. As shown in additional File 1, clear-cut elevated E-cadherin levels confirm the applied CDE procedure. Because a non-ambiguous oval cell marker is not available we displayed oval cells by both an anti-pan cytokeratin antibody, which stains biliary cells and oval cells [17] and by an anti-E-cadherin antibody which stains periportal hepatocytes, biliary cells and oval cells (Figure 1). The positive immunoreactivity was compared to an anti-M-Pk antibody staining (Rockland, USA) which was reported to detect oval cells as well [2], but we found nearly all sinusoidal cells positively marked (Figure 1). We confirmed this result using two further antibodies, which specifically recognize the M2-Pk epitope (clone DF4 and rabbit anti-M2-Pk, Table 1). Both antibodies also stained nearly all sinusoidal cells (see additional File 2). Only smooth muscle cells of the vessels were ambiguously labelled.

**Table 1:** Antibodies.

Antibody	Supplier/source	Dilution
Rat-anti-mouse CD31(PECAM-1)	BD Pharmingen	1:100
Rat-anti-mouse F4/80 (Clone A3-1)	Serotech	1:50
Rabbit-anti-cow-cytokeratin	DAKO	1:500
Rabbit-anti-cow-GFAP	DAKO	1:500
Goat-anti-rabbit-pyruvate kinase	Rockland incorporation	1:500-1:1,000
Mouse-anti-human pyruvate kinase (Clone DF 4)	Schebo Biotech AG	1:50
Rabbit-anti-human-M2-Pk	Cell Signaling	1:100
Chicken-anti-vimentin	Chemicon	1:5,000
Mouse-anti-vimentin S82		1:100
Rat-anti-BrdU	Serotech	1:50
Mouse-anti-human-E-cadherin	BD Transduction laboratories	1:100
Mouse-anti-rat-Nestin (Clone Rat-401)	Chemicon	1:100
Anti-alpha-smooth muscle actin (Clone 1A4)	SIGMA	1:100
Mouse-anti-human-N-cadherin	BD Transduction laboratories	1:100
Rabbit-anti-mouse-LI-cadherin	Gift from Dr. R. Geßner	1:1,000

**Figure 2:**
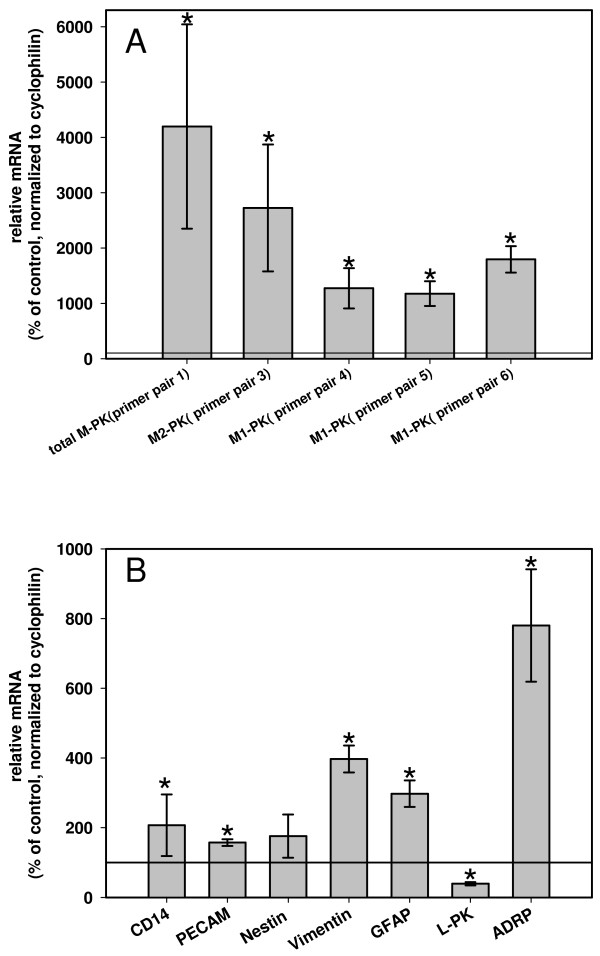
**Quantification of biomarkers in liver extracts of CDE treated mice**. Q-RT-PCR of total M-Pk, M1-Pk and M2-Pk with different primer pairs as indicated (A) and Q-RT-PCR of ADRP, a marker for lipid deposition in hepatocytes, L-Pk (exclusively expressed in hepatocytes), GFAP (classical marker of HSCs), vimentin (common marker of Kupffer cells, SECs, activated HSCs and fibroblasts), nestin (HSC marker), PECAM (CD31, marker for endothelial cells) and CD14 (cell surface marker of monocytes/macrophages like Kupffer cells) (B). Six treated mice were compared to six untreated age-matched mice. Reference line represents means in untreated mice set 100%. Statistical significant differences P < 0.05 (Mann Whitney ranks sum test) are indicated by an asterisk.

As expected, M2-Pk staining in CDE livers was more intense than in control livers. We validated the gain of M-Pk expression by Q-RT-PCR with different primer pairs, which amplify either both splice forms of M-Pk (primer pair 1; Table 2) or only M2-Pk (primer pair 3; Table 2) or M1-Pk (primer pairs 4, 5 and 6; Table 2) (Figure 2A). The identity of mouse M1-Pk was determined by sequencing of partial cDNA clones (M-Pk-up and M-Pk-down primer; additional File 3) derived from mouse heart, because this tissue is known to express solely M1-Pk. A strong up-regulation of both splice variants in livers of CDE treated mice was detected (Figure 2A).

**Table 2:** Primers.

	Upper primer	Lower primer	Accession number
Adipophilin	ccctgtctaccaagctctgc	cgatgcttctcttccactcc	NM_007408
L-Pk	ttctgtctcgctaccgacct	cctgtcaccacaatcaccag	NM_013631
GFAP	cacgaacgagtccctagagc	atggtgatgcggttttcttc	NM_012773
Vimentin	atgcttctctggcacgtctt	agccacgctttcatactgct	NM_011701
Nestin	gatcgctcagatcctggaag	gagaaggatgttgggctgag	NM_016701
PECAM1(CD31)	tgcaggagtccttctccact	acggtttgattccactttgc	NM_008816
CD14	ctgatctcagccctctgtcc	gcttcagcccagtgaaagac	NM_009841
Cyclophilin	aagactgaatggctggatgg	ttacaggacattgcgagcag	NM_008907
E-cadherin	tgctgattctgatcctgctg	ggagccacatcatttcgagt	NM_009864
N-cadherin	ctgggacgtatgtgatgacg	ggattgccttccatgtctgt	NM_007664
LI-cadherin	cctgaagcccatgacattct	ccgctcttgtttctctgtcc	NM_019753
M-Pk-pair 1	gcatcatgctgtctggagaa	gtaaggatgccgtgctgaat	NM_011099
M-Pk pair 3	tcgaggaactccgccgcctg	gtaaggatgccgtgctgaat	NM_011099
M-Pk pair 4	cagacctc atggaggcca tgg	gtaag gatgccgtgctgaat	Heart cDNA and NM_011099
M-Pk-pair 5	tgtttagcagcagctttg	ctatcattgccgtgactcga	Heart cDNA and NM_011099
M-Pk-pair 6	caccgtctgctgtttgaaga	ctatcattgccgtgactcga	Heart cDNA and NM_011099

Both, the elevation of M1-Pk and M2-Pk on RNA level and the increase of M-Pk positive cells point to expansion of sinusoidal cells due to CDE diet. Therefore, it was necessary to analyse the expression levels of known marker proteins of sinusoidal liver cells to prove which type of cells enriches due to CDE conditions. Two possibilities can be expected. In the case of sole enrichment of oval cells the M2-Pk elevation would exclusively be attributed to oval cells and vice versa the increase of M2-Pk under CDE diet might be considered as a marker of oval cell enrichment. But in the case of enrichment of other cell types during CDE diet and simultaneous expression of M2-Pk in these cell types, the enzyme is ultimately disqualified for being oval cell specific.

### Altered marker protein expression of sinusoidal liver cells indicates expansion of oval cells and HSCs under CDE diet

Expression levels of different published markers of sinusoidal cells (Table 3) were determined in CDE livers by Q-RT-PCR and compared to hepatocytic markers L-Pk and adipophilin, an indicator of fatty liver induction [18] (Figure 2B). As expected, we found a 2.5 fold reduced expression of L-Pk and a 7.8 fold induction of adipophilin in livers of CDE treated mice. The mRNA levels of all biomarkers of sinusoidal cells were up-regulated. Surprisingly, also an increase of GFAP was detected. Actually, GFAP is considered a marker of quiescent HSCs and CDE diet is regarded a fibrotic condition that should direct to activation and transdifferentation of HSCs into extracellular matrix producing myofibroblasts. This process is accompanied by an expression switch from GFAP to alpha smooth muscle actin (SMA). In this context a down-regulation of GFAP expression was expected. The observed elevation of GFAP expression also contrasts to the regular increase of two other activation markers of hepatic stellate cells, nestin and vimentin.

**Table 3:** Marker of liver cell types.

Protein	Cell type	Reference
ADRP	Hepatocytes Induction of fatty liver	[18]
L-Pk	Hepatocyte specific pyruvate kinase	[7]
GFAP	Quiescent hepatic stellate cells	[35]
Vimentin	Activated hepatic stellate cells	[33]
	Fibroblasts	[44]
	Sinusoidal endothelial cells	[34]
	Kupffer cells	[45]
Nestin	Activated hepatic stellate cells	[33]
PECAM(= CD31)	Activated defenestrated sinusoidal endothelial cells, endothelial cells of vessels	[38]
CD14	Macrophages and monocytes	[46]

**Figure 3:**
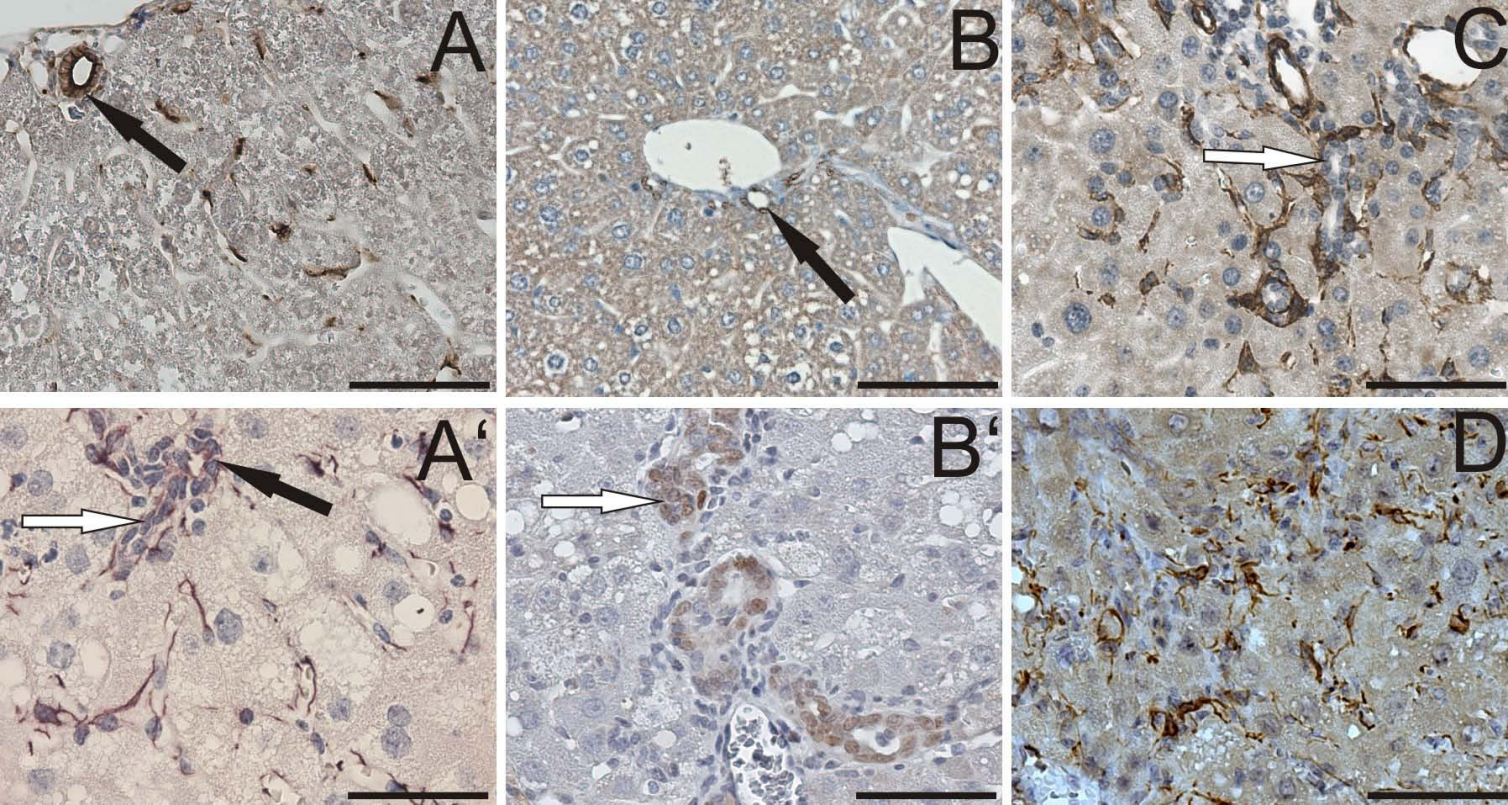
**Zonal differences of GFAP and GFAP-reporter expression in control and CDE treated mice in contrast to alpha-smooth muscle actin**. Immunohistochemistry of GFAP in liver sections of control (A) and CDE treated mice (A'). In B and B' the reporter enzyme Cre-recombinase has a nuclear localisation and was therefore used to demonstrate GFAP-promoter activity in CDE treated mice (B') compared to controls (B). HSCs are identifiable by their long, slender GFAP positive appendages. Biliary cells (black arrows) are also decorated with GFAP respectively express the Cre reporter. Under CDE conditions a third cell type, oval cells (brown, white arrows), express GFAP. The expression pattern of GFAP and GFAP-reporter in the periportal region of liver lobulus (A', B') is completely different from that in the pericentral region (D), (Cre in pericentral region is not shown, because there was no staining). Oval cell clusters, identifiable by their ductular formation, are surrounded by alpha-smooth muscle positive cells (C).

On histological level, we found a sophisticated expression pattern of GFAP in CDE livers compared to control ones (Figure 3). Firstly, a remarkable increase of GFAP positive HSCs in pericentral and midzonal region in CDE livers was detected (Figure 3D). Secondly, there was a quite variable positive staining of biliary cells in control livers and a distinct slight GFAP-positive staining of biliary cells and oval cells periportally in CDE livers (Figures 3A, A'). Vice versa GFAP positive cells with long appendices were only rarely seen periportally excluding any substantial enclosure of oval cells, which were instead surrounded by SMA-positive myofibroblasts as already reported previously [4] and shown here (Figure 3C). GFAP staining in biliary cells (cholangiocytes) was already demonstrated previously [19], whereas the GFAP expression in mouse oval cells is a new finding and potentially opens a link to HSCs. The identity of an oval cell specific GFAP signal was subsequently further verified by examining liver tissue of transgenic mice that express Cre-recombinase driven by a GFAP-promoter (GFAP-Cre-mouse). Because Cre-recombinase (Cre) is a recombinant protein, any cross reactivity with antibodies directed against endogenous mouse protein is prevented. Its nuclear localization allows a clear discrimination of cell types. We detected Cre-positive biliary cells in untreated mice and Cre-positive biliary cells and oval cells in CDE treated GFAP-Cre-mice (Figure 3B, B').

The immunohistological examination of livers of CDE treated mice relative to the other markers listed in Table 3 shows that Kupffer cells (positively stained by anti-F4/80-antibody), vimentin-, PECAM (CD31)- and nestin-positive cells expand in addition to GFAP-positive cells in CDE liver sections (additional File 4). To exclude a misinterpretation due to the mixed genetic background of the mice used in our study, we also included paraffin embedded tissue of a former CDE study using C57Bl/6 mice [5] and confirmed our results (data not shown).

### Oval cells, HSCs and Kupffer cells proliferate due to CDE diet and likewise rapidly growing liver related cell lines express M2-Pk

**Figure 4:**
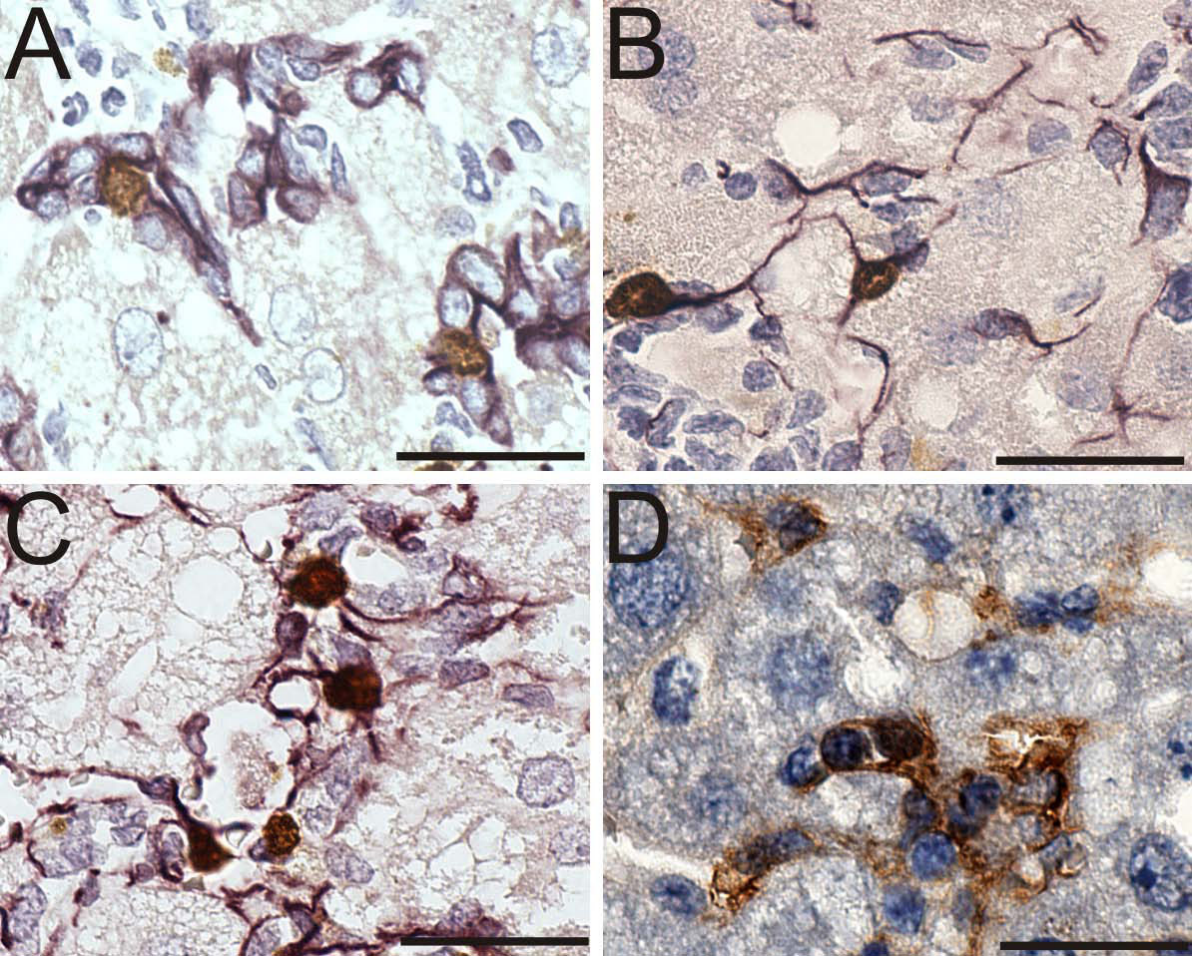
**Expansion of oval cells and sinusoidal cells under CDE conditions is proliferative**. Double-immunohistochemistry of BrdU with cytokeratin (A), BrdU with GFAP (B), BrdU with vimentin (C) and BrdU with F4/80 (D). In A, B and C, BrdU-positive nuclei are labelled in brown and the corresponding biomarkers in purple. In (D) BrdU-positive nuclei are labelled in purple and the corresponding Kupffer cell marker (F4/80) in brown. Nuclei were counterstained with hematoxylin (blue). Bars = 50 μm.

M2-Pk is commonly known to elevate in rapidly growing cells. Firstly, we tested the proliferative state of distinct sinusoidal cell populations by double labelling experiments combining BrdU-staining with biomarker staining in liver sections of CDE treated mice (Figure 4). BrdU positive cells occur in clusters pointing to clonal expansion. As expected, BrdU/cytokeratin (oval cells) double-positive cells were restricted to the periportal area (Figure 4A), whereas BrdU/strong GFAP double positively labelled HSCs and BrdU/vimentin double-positive cells were found almost exclusively in the pericentral region. In contrast, BrdU/F4/80 (Kupffer cells) double-positive cells were uniformly distributed over the whole lobule, but enriched in clusters around perished hepatocytes (Figure 4D). No BrdU/CD31 double positive cells were detected, though increased expression of CD31 was determined by Q-RT-PCR and *in situ*. This fact points to a rise of CD31 expression in existing sinusoidal endothelial cells (not shown).

Secondly, we examined rapidly growing mouse liver related cell lines for their expression of M-Pk and compared it to primary hepatocytes and freshly isolated sinusoidal cells. We included into our study oval cell lines OVUE867 and 265 [20], the monocyte/macrophage cell line RAW264.7 (DSMZ, Braunschweig, Germany), the hepatic stellate cell line HSC-Mim 1-4 [21], the liver tumor cell line Hepa 1C7 (DSMZ, Braunschweig, Germany), as well as primary sinusoidal endothelial cells (SECs) and primary sinusoidal cells both derived from freshly isolated mouse liver of control mice. Obtained RT-PCR products were cloned and at least five clones from every cell type were sequenced. Clones from cell lines were 100% M2-Pk homologous. Seventy% of the sequenced clones from primary SECs and sinusoidal cells were from M2-Pk type and 30% of the clones displayed M1-Pk sequence. Probably, the M1-Pk signal is due to remaining cell contamination of primary cells with smooth muscle cells of liver vessels.

### M2-Pk colocalises with most sinusoidal cell populations

**Figure 5:**
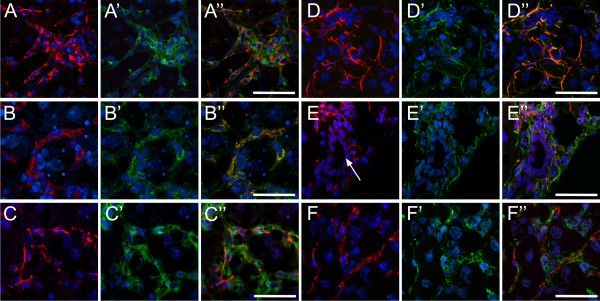
**Confocal laser scanning microscopy of M2-Pk and biomarkers of sinusoidal liver cells**. Double immunofluorescence of M2-Pk (green, A', B', C') with F4/80 (red, A), with GFAP (red, B) and with vimentin (red, C). Merged images are shown in A'', B'' and C'', respectively. Colocalization of GFAP (red, D, E) with vimentin in a pericentral (green, D') and in a periportal (green, E') region is shown in D'' and E'', respectively. Faint red fluorescence of the membranes of biliary cells is indicated by the white arrow in E. Colocalization of CD31 immunoreactivity (red, F) with vimentin (green, F') is shown in F''. Immunofluorescence stainings were recorded by Confocal Laser Scanning microscopy. Bar = 20 μm.

We analysed double fluorescence stainings of M2-Pk (antibody DF-4, Table 1) with markers of sinusoidal cells using laser scanning microscopy to attribute the M2-Pk signal to the appropriate cell type (Figure 5). M2-Pk colocalized with F4/80 (Kupffer cell marker, Figure 5A), GFAP (HSC marker, Figure 5B) and vimentin in pericentral and midzonal regions (Figure 5C). Double fluorescence of anti-vimentin with anti-CD31 demonstrates that SECs belong to the vimentin positive cell type (Figure 5F).

Double fluorescence of vimentin with GFAP assigns pericentral/midzonal activated HSCs to the mesenchymal cell pool (Figure 5D), which is well separated from the faintly GFAP positive periportal cell pool (Figure 5E). There was no overlapping expression of M2-Pk with smooth muscle actin (not shown).

### Cell adhesion in CDE livers

Both, loss of hepatocytes and the integration of stem cells in liver tissue require a rearrangement of cell-cell contacts in liver tissue. These contacts are mainly established by adherens junctions that are formed by cadherins. Like other authors [4] we also found E-cadherin overexpressed in CDE livers (Figure 1 and additional File 1), but identified additionally P-cadherin and LI-cadherin elevated (additional File 1). Because LI-cadherin was the most up-regulated cadherin and is the embryonal mouse liver form it was expected that this cadherin is related to oval cells because of their stem cell character. However, immunostaining of liver sections of CDE-treated mice shows clearly that this embryonal form is re-expressed by hepatocytes (additional File 1).

## Discussion

The two well established consequences of CDE diet in mouse liver, enrichment of oval cells and up-regulation of M-Pk [2,13-15], were re-evaluated in our study and must be interpreted from a new perspective. Our results advise to discuss these two phenomena on two independent levels.

Firstly, an increase of M-Pk in liver of CDE treated mice reflects the sum of elevated M1- and M2-Pk. For the first time, the two forms in mouse liver extracts under CDE conditions were differentially studied. The quantification of M-Pk with a PCR method not distinguishing between the two forms [6] can not be accepted to be a specific signal of oval cells, because our *in vitro *data clearly show that oval cells express only M2-Pk. This result is of special interest in time slot studies, because it was shown recently that a myofibroblastic expansion precedes the oval cell proliferation in CDE diet [4]. Accordingly, at different time points of CDE diet the fraction of M1- and M2-type may vary considerably.

Secondly, M2-Pk reflects the activation of both oval cells and sinusoidal cell types as revealed by our *in situ *results. Thus, our results verify for the mouse the earlier findings in rats that endothelial cells, biliary cells, Kupffer cells [7,10] and HSCs [9] express M2-Pk. Furthermore, also infiltrating immune cells may contribute to M2-Pk expression demonstrated by our *in vitro *results. In addition to the early expansion of myofibroblasts [4], we definitely show that at least HSCs and Kupffer cells expand due to proliferation in CDE livers and M2-Pk is preferentially expressed in exactly the cells with high DNA synthesis. Therefore, M2-Pk should not longer be considered a specific oval cell marker.

A new and remarkable result of our study is the GFAP expression pattern in livers of CDE treated mice. GFAP is commonly used to detect HSCs, since it specifically detects this cell type in normal rat liver [22]. We observed GFAP expression in three cell types, in HSCs and biliary cells in all liver samples and in oval cells under CDE conditions. The GFAP expression in epithelial cells of biliary ducts was recently also detected by others [19] and a TGF-β dependent up-regulation of GFAP was demonstrated in cultured rat oval cells [23]. If GFAP is expressed in biliary cells as well as in HSCs, then any fate mapping based on GFAP promoter activity, as recently used for tracing the source of oval cells [19], becomes less convincing. Moreover, we detected in GFAP-Cre mice no nuclear signal of Cre-reporter in HSCs but only in biliary cells and oval cells. This is exactly the localization, which was reported from various GFAP promoter reporter mice [24,25]. It is remarkable that GFAP expression of oval cells fits in the list of other published oval cell markers that share their expression with one of the epithelial cell types of liver. For example, the A6 antigen [26] and cytokeratins are also expressed in cholangiocytes, and E-cadherin is found in both, portal hepatocytes and cholangiocytes [16]. Even the stem cell marker CD133 used for defining a subpopulation of HSCs [27] was also found in oval cells [28]. This intercellular sharing of subsets of surface antigens among cells of epithelial and mesenchymal morphology suggests that EMT (and possible MET) might play a much greater role in liver regeneration under toxic conditions than previously thought. Thus, solving the mystery of how liver regeneration from stem cells and progenitor cells is achieved seems to remain an ongoing challenge waiting for more sophisticated cell biological techniques. As we state herein biomarkers may help in this endeavour only, if their expression is carefully studied under the specific conditions used.

A second important aspect of GFAP expression is linked to its strong up-regulation in CDE mouse livers. As shown herein this is due to enhanced proliferation of HSC in the midzonal/pericentral region. Similarly, up-regulation of GFAP was shown in injured human [29], rat [30], and mouse liver [31] and seems comparable to the complex reaction of "gliosis" in brain as a response to many injuries of CNS. Gliosis also includes both proliferation and hypertrophy of GFAP expressing cells [32]. Two other markers, nestin and vimentin, were expressed by activated HSCs [33] a finding confirmed herein for the activation of GFAP positive HSCs (all GFAP positive HSCs coexpressed vimentin) under CDE conditions.

For the first time, the proliferation of midzonal and pericentral located HSC populations was shown. This is also important for considering the origin of myofibroblasts, which play a central role in matrix synthesis and remodelling during oval cell expansion. Like others [4,15] we also detected a strong up-regulation of SMA positive cells in CDE livers. Interestingly, periportal SMA positive cells co-expressed vimentin, a protein actually synthesized in fibroblasts [34], suggesting their origin from periportal (myo-)fibroblasts rather than from HSCs, since co-expression of GFAP, a characteristic for the transdifferentiation into myofibroblasts demonstrated *in vitro *[35,36] but not *in vivo*, was rarely detectable. Even though we might have missed such an event in an early phase after exposure to CDE, it is remarkably that the above mentioned activation of HSC persists even after two weeks. Thus, HSCs seem to have other functions than transdifferentiation to myofibroblasts as it was discussed in a recent study using a rat oval cell model [37].

Up-regulation of CD31 (PECAM) in livers of CDE treated mice is another new finding of this study. The lack of any BrdU/CD31 co-expression points to an increase of CD31 in SECs. In untreated livers CD31 positive cells were hardly detected, whereas up-regulation seems to be associated with dedifferentiation of SECs into a defenestrated endothel during pseudocapillarization due to fibrotic processes [38] which also occur under CDE conditions [4].

The impact of re-expression of LI-cadherin in adult mouse liver during CDE diet is still unclear and currently under investigation in double knock-out mice for LI and E-cadherin in our group. Possibly, re-expression of LI-cadherin, an embryonal marker of mouse liver [39], prevents the dissociation of cellular connections on sites of insufficient expression of E-cadherin.

## Conclusions

The present study clearly shows that in mouse liver M2-Pk is expressed in nearly all cells of hepatic sinusoid. Undisputable CDE diet leads to an up-regulation of M-Pk, but this rise is the summation of M1- and M2-Pk. The elevation should no longer be misinterpreted as a specific oval cell response. Under CDE conditions GFAP expressing cells expand in a zonal specific pattern. Pericentral GFAP positive cells seem to present an activated cell type. Periportal oval cells express GFAP, a common HSC marker. Therefore, this marker does not seem suitable for tracing progenitors of hepatocytes under CDE conditions.

## Methods

### Animals

GFAP-tTA mice (B6.Cg.Tg(GFAP-tTA)110Pop/J, Jacksons Laboratory, Bar Harbor, USA) were intercrossed with p_tet_Cre mice (LC1, [40]) resulting in double transgenic mice expressing Cre-recombinase by GFAP promoter driven tTA expression (GFAP-Cre-mice). Mice of mixed genetic backround (DAB/C57Bl/6) and GFAP-Cre mice were given a CDE diet over 14 days. Cholin deficient animal chow without addition of methionine (Altromin, Lage, Germany) was provided ad libitum and drinking water was replaced by 0.165% ethionine solution (TCI, Europe, Zwijndrecht, Belgium) and was also given ad libitum. Animal experiments were carried out in accordance with the European Council Directive of 24 November 1986 (86/609/EEC) and were approved by local authorities. 10 week old mice of mixed genetic background (DBA/C57Bl/6) and GFAP-Cre mice were used as controls. All mice received a single i.p. injection of BrdU (10 mM, 1 ml per 100 g bodyweight) 2 h before killing.

### Histology and immunohistochemistry

Liver samples were either quick-frozen in liquid nitrogen and stored at -80°C or fixed in 4% paraformaldehyde and routinely embedded in paraffin. Frozen liver samples were used for PECAM1 immunohistochemistry and were processed as described [16]. For all other antibodies (Table 1) and hematoxylin-eosin (HE) staining 2 μm paraffin sections were used and processed as described [16] Antigen-antibody complexes were detected by peroxidase- or Cy-2/3-conjugated secondary antibodies as previously described [41,42]. Similarly processed liver slides where the primary antibody was omitted were used as negative controls. Monoclonal mouse antibodies were used together with the Vector M.O.M. Immunodetection Kit (Vector Laboratories, CA, USA) to avoid a cross-reactivity of secondary antibodies with endogeneous immunoglobulins of mouse tissue.

For detection of Kupffer cells (the liver specific macrophages), the anti-F4/80 antibody was used instead of an antibody against the macrophage/monocyte marker CD14.

### Isolation of liver cells and cell culture

Hepatocytes were isolated using an *in vitro *perfusion technique [43]. Liver was perfused with calcium free buffered saline and subsequently with collagenase (1 mg/ml, 240 U/mg, Biochrom AG, Berlin, Germany). Cell suspension was centrifuged thrice at 70 × g, 5 min. Sinusoidal cells were isolated by perfusing liver consecutively with calcium free buffered saline, pronase (1 mg/ml) and collagenase (1 mg/ml) for 10 min each. Cell suspension was centrifuged twice at 70 × g disposing the hepatocytes and twice at 250 × g for washing and collecting sinusoidal cells. Cells were re-suspended and either undergone RNA isolation or incubated with anti-CD146 antibody linked to magnetic beads according to the suppliers recommendation (Miltenyi Biotec GmbH, Bergisch Gladbach, Germany). CD146 positive SECs were eluted after magnetic separation. After two washings RNA was extracted.

### Isolation of RNA and quantitative real time RT-PCR (Q-RT-PCR)

Total RNA was isolated using the PeqGOLD RNA Pure isolation system (Peqlab, Erlangen, Germany). Quality of RNA was assessed by electrophoresis in denaturing formaldehyde agarose gels and purity was estimated by ratio A260/280 nm spectrophotometrically. Concentration was adjusted to 0.5 mg/ml. RT-PCR for real time quantification was performed as previously described [42] using primers listed in Table 2. RNA sample load was normalized using amplifications with the housekeeping gene cyclophilin. Standard curves of serial dilutions from total RNA were used for transforming the ct-values in concentration values depicted as arbitrary units.

For primer design of total M-Pk and M2-Pk the RNA sequence [Genbank: NM_011099] was used. For this purpose we amplified M-Pk cDNA, generated from RNA of freshly isolated liver cells of control mice and cultivated cell lines, with the M-Pk-up and M-Pk-down primers (additional File 3).

### Statistical analysis

All data are expressed as mean ± SEM. Statistical analysis was performed by Student's t-test or Mann Whitney Ranks sum Test using Sigma plot 11 (SSP Science, Chicago, IL, USA). The accepted level of significance was set at P < 0.05.

## Competing interests

The authors declare that they have no competing interests.

## Authors' contributions

EU, JB and UU acquired, analysed and interpreted the data. JG made the confocal laser scanning microscopy and edited the figures. EU wrote the first draft of the manuscript and UU and RG co-wrote the final version. All authors have read and approved the manuscript.

## Supplementary Material

Additional file 1 **Expression of cadherins confirms effectiveness of CDE diet conditions**. A Q-RT-PCR screen (A) verified the over-expression of E-cadherin in CDE diet mice compared to untreated controls. Remarkably, LI-cadherin the embryonal expressed liver cadherin was even strongerly increased. Statistically significant differences P < 0.05 (Mann Whitney ranks sum test) are indicated by an asterisk. Immunohistochemistry with anti-LI-cadherin antibody (B, B') demonstrates the re-expression of LI-cadherin in hepatocytes of CDE treted mice (B'). LI-cadherin is not detectable in normal adult mouse liver (B). Bar = 50 μm. Click here for file

Additional file 2 **M2-Pk demonstration in livers of CDE treated mice**. Immunohistochemistry with anti-M2-Pk (DF4, Schebo GmbH, Germany, A) and anti-M2-Pk (Cell Signaling, USA, A') Smooth muscle cells are indicated by white arrows. Bar = 50 μm. Click here for file

Additional file 3 **cDNA Sequence of M-Pk and primers for M-Pk quantification and sequencing**. M2-Pk and M1-Pk have the same sequence except for exon 9. Exon 8 and exon 10 are highlighted in gray. The first line shows the shared sequence of M1- and M2-Pk and the second line shows the different sequence of M1-Pk in exon 9. Primers used for sequencing of RT-PCR-products of cell lines and isolated cells were marked M-Pk-up and M-Pk-down. For real time quantification of total M-Pk primer pair 1 (M-Pk-f1 (gcatcatgctgtctggagaa and M-Pk-down) was used. M2-Pk was quantified with primer pair 3 (upper de Luis-primer and M-Pk-down). M1-RT-PCR was done with primer pair 4 (M1-f-neu and M-Pk-down), primer pair 5 (M1-rev-neu and M-Pk-forward) and primer pair 6 (M1-f-512 up and M1-down 715). Primers used by authors Fleig et al 2007 are indicated. These primers are lying in exon 11 and therefore detect both isoforms forms together. Sequence of M2-Pk (NM_011099) was fetched from Entrez Nucleotide database on NCBI http://www.ncbi.nlm.nih.gov. Click here for file

Additional file 4 **Number of cells of hepatic sinusoids raised in CDE treated mice**. Cells of hepatic sinusoids were depicted by immunohistochemistry with an anti-F4/80 antibody (Kupffer cell, A, A'), an anti-vimentin-antibody (mesenchymal cells, B, B'), an anti-nestin antibody (activated HSCs, C, C') and an anti-CD31 (marker of defenestrated endothelial cells, D, D'). Bar = 50 μm. Click here for file
