# Supplementary figures and images for: Response of sinusoidal mouse liver cells to choline-deficient ethionine-supplemented diet
Source: Comp Hepatol. 2010 Oct 13;9:8. doi: 10.1186/1476-5926-9-8 (PMC2964607; doi:10.1186/1476-5926-9-8)

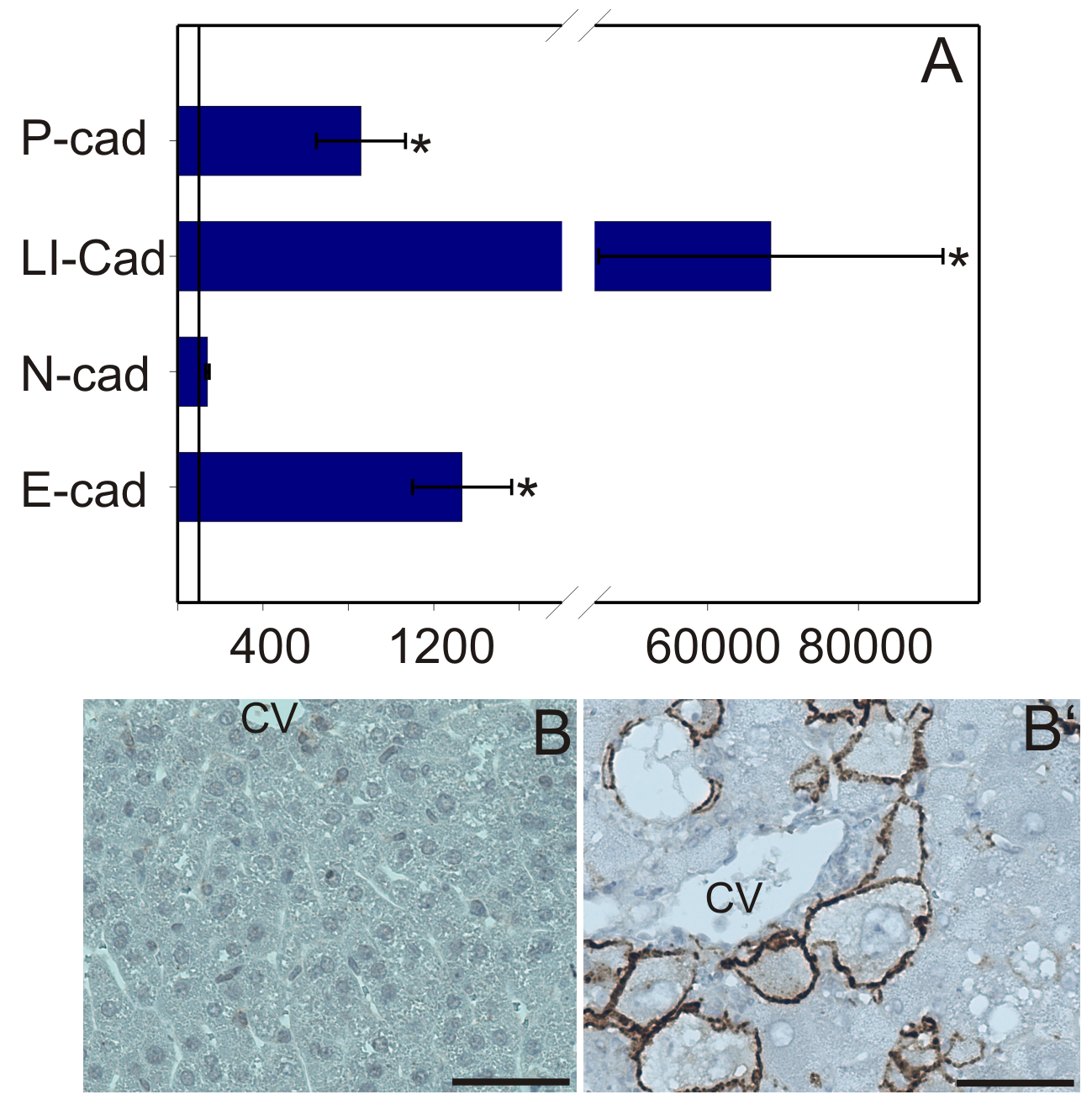

Supplement: Additional file 1 — Expression of cadherins confirms effectiveness of CDE diet conditions. A Q-RT-PCR screen (A) verified the over-expression of E-cadherin in CDE diet mice compared to untreated controls. Remarkably, LI-cadherin the embryonal expressed liver cadherin was even strongerly increased. Statistically significant differences P < 0.05 (Mann Whitney ranks sum test) are indicated by an asterisk. Immunohistochemistry with anti-LI-cadherin antibody (B, B') demonstrates the re-expression of LI-cadherin in hepatocytes of CDE treted mice (B'). LI-cadherin is not detectable in normal adult mouse liver (B). Bar = 50 μm. [file 1476-5926-9-8-S1.TIFF]

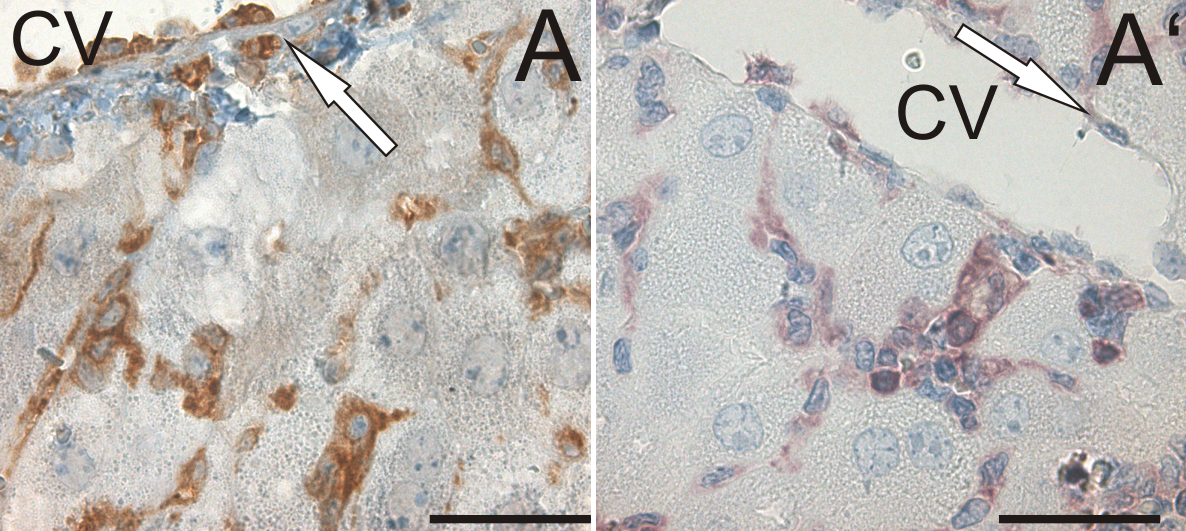

Supplement: Additional file 2 — M2-Pk demonstration in livers of CDE treated mice. Immunohistochemistry with anti-M2-Pk (DF4, Schebo GmbH, Germany, A) and anti-M2-Pk (Cell Signaling, USA, A') Smooth muscle cells are indicated by white arrows. Bar = 50 μm. [file 1476-5926-9-8-S2.TIFF]
