# Supplementary material for: Response of sinusoidal mouse liver cells to choline-deficient ethionine-supplemented diet
Source: Comp Hepatol. 2010 Oct 13;9:8. doi: 10.1186/1476-5926-9-8 (PMC2964607; doi:10.1186/1476-5926-9-8)
Supplement: Additional file 3 — cDNA Sequence of M-Pk and primers for M-Pk quantification and sequencing. M2-Pk and M1-Pk have the same sequence except for exon 9. Exon 8 and exon 10 are highlighted in gray. The first line shows the shared sequence of M1- and M2-Pk and the second line shows the different sequence of M1-Pk in exon 9. Primers used for sequencing of RT-PCR-products of cell lines and isolated cells were marked M-Pk-up and M-Pk-down. For real time quantification of total M-Pk primer pair 1 (M-Pk-f1 (gcatcatgctgtctggagaa and M-Pk-down) was used. M2-Pk was quantified with primer pair 3 (upper de Luis-primer and M-Pk-down). M1-RT-PCR was done with primer pair 4 (M1-f-neu and M-Pk-down), primer pair 5 (M1-rev-neu and M-Pk-forward) and primer pair 6 (M1-f-512 up and M1-down 715). Primers used by authors Fleig et al 2007 are indicated. These primers are lying in exon 11 and therefore detect both isoforms forms together. Sequence of M2-Pk (NM_011099) was fetched from Entrez Nucleotide database on NCBI http://www.ncbi.nlm.nih.gov. [file 1476-5926-9-8-S3.PDF]

Mus musculus pyruvate kinase, muscle (Pkm2), mRNA
ACCESSION NM_011099

```
   1 gtccgctcta ggtatcgcag caggaaccga agtacgcccg aggacttcag gaaccatgcc
  61 gaagccacac agtgaagcag ggactgcctt cattcagacc cagcagctcc atgcagccat
 121 ggctgacacc ttcctggaac acatgtgccg cctggacatt gactctgccc ccatcacggc
 181 ccgcaacact ggcatcattt gtaccattgg gcctgcttcc cgatctgtgg agatgctgaa
 241 ggagatgatt aagtctggaa tgaatgtggc tcggctgaat ttctctcatg gaacccatga
 301 gtaccatgca gagaccatca agaatgtccg tgaagccaca gaaagctttg catctgatcc
 361 cattctctac cgtcctgttg cggtggctct ggatacaaag ggacctgaga tccggactgg
 421 actcatcaag ggcagcggca ccgctgaggt ggagctgaag aagggagcca ctctgaagat
 481 caccctggac aacgcttaca tggagaagtg tgacgagaac atcctgtggc tggactacaa
 541 gaacatctgc aaggtggtgg aggtgggcag caagatctac gtggacgatg ggctcatctc
 601 actgcaggtg aaggagaaag gcgctgactt cctggtgacg gaggtggaga atggtggctc
 661 cttgggcagc aagaagggcg tgaacctgcc gggcgctgct gtggatctcc ccgctgtgtc
 721 ggaaaaggac atccaggacc tgaagtttgg ggtggagcag gatgtggaca tggtgtttgc
 781 atctttcatc cgcaaggcag ccgacgtgca tgaagtcagg aaggtgctgg gagagaaggg
 841 caagaacatc aagatcatca gcaaaatcga gaaccatgaa ggcgtccgca ggtttgatga
 901 gatcttggag gccagtgatg ggatcatggt ggctcgtggt gacctgggca ttgagattcc
 961 tgcagagaag gtcttcctgg ctcagaagat gatgatcggg cgatgcaacc gagctgggaa
                                  Exon8
1021 gcctgtcatc tgtgccacac agatgctgga gagcatgatc aagaagccac gccccacccg
1081 tgctgaaggc agtgatgtgg ccaatgcagt cctggatgga gcagactgca tcatgctgtc
     M-PK-up                                                   Exon 9
1141 tggagaaaca gccaaggggg actaccctct ggaggctgtt cgcatgcagc acctgattgc
                                               Upper de Luis      atag
1201 ccgagaggca gaggctgcca tctaccactt gcagctattc gaggaactcc gccgcctggc
     ctcgggaggc tgaggcagcc atgttccacc gtctgctgtt tgaagagctt gtgcgagcct
                                   M1-f-512-up
1261 gcccattacc agcgacccca cagaagctgc cgccgtgggt gccgtggagg cctccttcaa
     ccagtcactc cacagacctc atggaggcca tggccatggg cagcgtggag gcctcttata
                M1-f-neu                                 Exon 10
1321 gtgctgcagt ggggccatta tcgtgctcac caagtctggc aggagtgctc accaagtggc
     agtgtttagc agcagctttg atagttctca cgg
       M1-rev-neu
1381 caggtaccgc cctcgggctc ctatcattgc cgtgactcga aatccccaga ctgctcgcca
                          M-down-715          M-PK-down
1441 ggcccatctg taccgtggca tcttccctgt gctgtgtaag gatgccgtgc tgaatgcctg
1501 ggctgaggat gtcgaccttc gtgtaaactt ggccatggat gttggcaagg cccgaggctt
1561 cttcaagaag ggagatgtgg tcattgtgct gaccgggtgg cgccctggct ctggattcac
             Fleig et al 2007
1621 caacaccatg cgtgtagtgc ctgtaccttg atggccctct ggagcccctc ttctagcccc
1681 tgtcccttcc cctcccctat cctttccatt aggccagcaa cgcttgtagt gctcactctg
1741 ggccatagtg tggcgctggt gggctgggac accagggaaa attaatgcct ctaaaacatg
1801 caatagagac cagctattat tcagggccct acctgagcca ggggtggagg aggaatgcag
                 Fleig et al 2007
1861 gactggaaac cctgacttta tcacagaagg gcggcagcat ctctgggctt tgcttctgta
1921 gaaagttgtc agaattccca gccctagcct ggagtcagga gacagcaaaa gagtaggggc
1981 tgagggtgtg gggcccaggg tcccagtgta gatgacgact tctggccctg gccctgacct
2041 gctttcccaa cagctttggc ctccccactt cttgtgcact ccacttctgt cactgcagac
2101 actccactct ccaccttgta ttctgcagag tctccaggcc tgttgctata gtgcccacct
2161 gaatgtcaat aaacagcagc tgaagcacct gtaaaaaaaa aaaaaaaaaa aa
//
```
